# Correlation of retinal vascular characteristics with laboratory and ocular findings in Fabry disease: exploring ocular diagnostic biomarkers

**DOI:** 10.1186/s13023-023-02932-x

**Published:** 2023-10-08

**Authors:** Migle Lindziute, Jessica Kaufeld, Karsten Hufendiek, Ingo Volkmann, Dorothee Brockmann, Sami Hosari, Bettina Hohberger, Christian Mardin, Carsten Framme, Jan Tode, Katerina Hufendiek

**Affiliations:** 1 University Eye Hospital, Hannover Medical School, Hannover, Germany; 2 Division of Nephrology, Center for Internal Medicine, Hannover Medical School, Hannover, Germany; 3 Department of Surgery, Friedrich-Alexander-University of Erlangen-Nuremberg, Erlangen, Germany; 4 Department of Ophthalmology, Friedrich-Alexander-University of Erlangen-Nuremberg, Erlangen, Germany

**Keywords:** OCT angiography, Fabry disease, Vessel area density, Vascular remodelling, Biomarkers

## Abstract

**Background:**

The goal of this study was to evaluate macular microvascular changes in patients with Fabry disease (FD) using optical coherence tomography angiography (OCTA) and to explore their correlation with laboratory and ocular findings.

**Methods:**

A total of 76 eyes (38 patients) and 48 eyes of 24 healthy controls were enrolled in this prospective study. Vessel Area Density (VAD) and Foveal Avascular Zone (FAZ) area were calculated on 2.9 × 2.9 mm OCTA images scanned with the Heidelberg Spectralis II (Heidelberg, Germany). VAD was measured in three layers: Superficial Vascular Plexus (SVP), Intermediate Capillary Plexus (ICP), and Deep Capillary Plexus (DCP). All scans were analyzed with the EA-Tool (Version 1.0), which was coded in MATLAB (The MathWorks Inc, R2017b). FAZ area was manually measured in full-thickness, SVP, ICP and DCP scans.

**Results:**

Average VAD in SVP, ICP and DCP was higher in Fabry disease patients than in controls (49.4 ± 11.0 vs. 26.5 ± 6.2, 29.6 ± 7.4 vs. 20.2 ± 4.4, 32.3 ± 8.8 vs. 21.7 ± 5.1 respectively, *p* < 0.001). Patients with cornea verticillata (CV) had a higher VAD in ICP and DCP compared to patients without CV (*p* < 0.01). Patients with increased lysoGb3 concentration had a higher VAD in DCP when compared to patients with normal lysoGb3 concentration (*p* < 0.04). There was no difference in VAD in patients with and without vascular tortuosity. However, a significantly higher VAD was observed in patients with vascular tortuosity compared to controls (*p* < 0.03).

**Conclusions:**

Increased lysoGb3 and VAD in DCP could be reliable biomarkers of disease activity. Cornea verticillata could be adopted as a predictive biomarker for VAD changes and disease progression. The combination of cornea verticillata and increased VAD may serve as a diagnostic biomarker for Fabry disease, however due to the discrepancies in VAD values in various studies, further research has to be done to address this claim.

## Background

Fabry disease (FD) is an X-linked disorder of glycosphingolipids that is caused by mutations of the alpha galactosidase (GLA) gene that codes for alpha galactosidase A, leads to dysfunction of many cell types and includes a systemic vasculopathy [1].

Reduced or absent activity of the enzyme α-galactosidase A results in progressive accumulation of glycolipids, primarily globotriaosylceramide (Gb_3_) and its deacylated form, globotriaosylsphingosine (lyso-Gb-3) in plasma and in a wide range of cells throughout the body [2]. This manifests as serious and progressive impairment of renal and cardiac functions [2, 3].

**Fig. 1:**
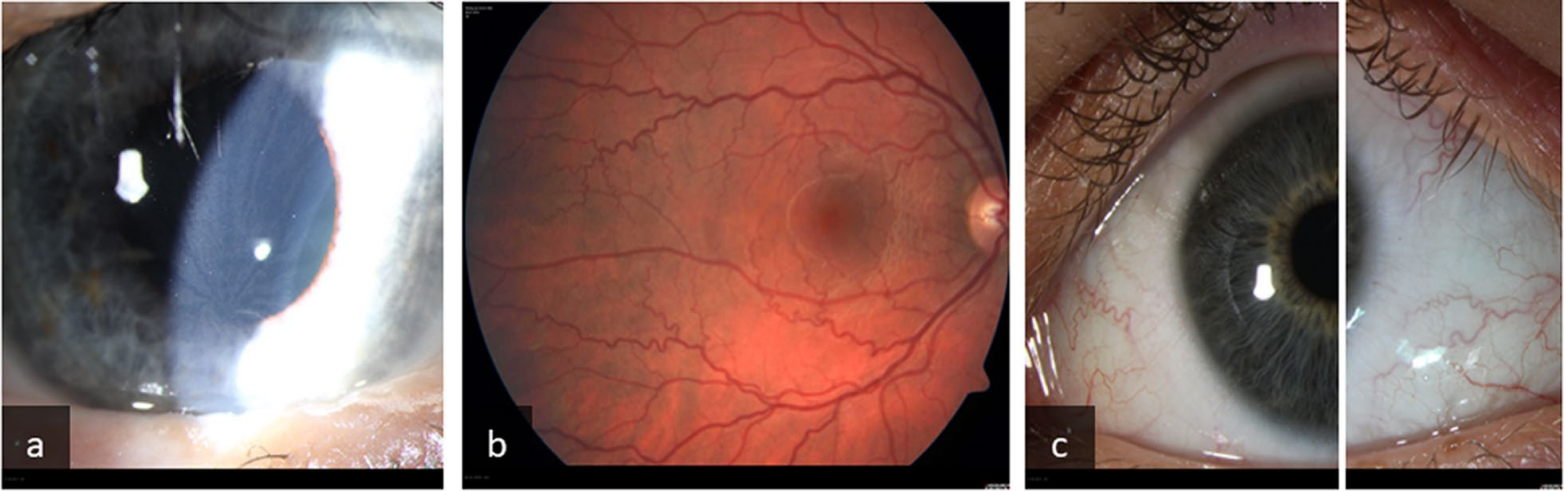
Ocular manifestations of Fabry disease: cornea verticillata (**a**), retinal vessel tortuosity (**b**) and conjunctival vessel tortuosity (**c**)

Additionally, some patients may experience angiokeratomas, peripheral neuropathy, pain, vestibular disease, hearing loss, gastrointestinal disturbance, cerebral vasculopathy, transient ischemic attacks and strokes [3, 4]. The most commonly reported ocular clinical sign of FD is cornea verticillata (CV) [5, 6]. Cornea verticillata (Fig. 1) may be an isolated finding in patients with Fabry's disease without other eye abnormalities, therefore it is the most reliable ocular marker of the disease [5]. Ophthalmological manifestations of FD also include anterior capsular or posterior subcapsular cataracts as well as vessel tortuosity, microaneurysms, and general dilation in conjunctival and retinal blood vessels (Fig. 1) [5–7].

When looking into the pathology of FD both small and large blood vessels are proven to be involved in the cerebral vasculopathy in Fabry disease [8]. However, the changes in the cerebral microcirculation are very difficult to visualize. Because of this OCTA is a very promising non-invasive tool in understanding more about microvascular changes in Fabry disease.

Plasma levels of lyso-Gb3, a Gb3 degradation product, can be used to evaluate accumulation of sphingolipids and have been shown to be more sensitive and specific then measuring Gb3 concentration in urine [9, 10]. It is speculated that plasma lyso-Gb3 could be promising biomarker of FD [9, 11]. Early diagnosis, evaluation of progression and management of FD could be improved by identifying more biomarkers that reflect activity and progression of the disease.

The aim of our study was to evaluate macular microvascular changes in Fabry disease patients using OCTA and correlating these changes with laboratory and ocular findings to evaluate their potential as biomarkers for activity and progression of the disease.

## Methods

### Study design, patient selection and examination

We conducted a prospective study of 76 eyes of 38 patients with Fabry disease and 48 eyes of 24 healthy controls. Patients were recruited through the nephrology department of Hannover Medical School, Germany from January 2017 to December 2021 and were genetically confirmed to have FD before enrolment in the study. The healthy controls were recruited from accompanying persons, visitors or students in the Department of Ophthalmology. The healthy controls did not have any pre-existing systemic or ocular conditions and received a ophthalmological examination to rule out any pathologies.

The study was conducted according to the Declaration of Helsinki and was approved by the Ethic Committee of Medical School Hannover, Germany (Ethics Approval Nr. 7687). Written informed consent was obtained from each participant before enrolment in the study.

All participants underwent a complete ophthalmological examination including best corrected visual acuity, slit lamp and fundus examination for ocular involvement of FD. OCTA images (2.9 × 2.9 mm) were obtained using Heidelberg Spectralis II (Heidelberg Engineering GmbH, Heidelberg, Germany; Acquisition Software Version 6.12.4.0).

Patients that suffered from other ophthalmological disorders (like age-related macular disease, diabetic retinopathy, retinal arterial or venous occlusions, other retinal vessel disorders, glaucoma, uveitis, dense cataracts, high grade myopia or hyperopia with a refractive error of more than 3.5 dpt) as well as incompliant patients or patients not willing or able to give informed consent were excluded from the study. Healthy controls were not included in the study if any ophthalmological disorders were diagnosed during our examination.

Cornea verticillata und vascular tortuosity were evaluated clinically by an experienced ophthalmologist during the examination of the patients. Systemic parameters such as LysoGb3 plasma concentration or GLA enzyme activity were evaluated.

### OCTA image processing and measurement of vessel area density (VAD) in the macular area

The quality of the segmentation in horizontal foveal OCT B scans in the OCTA examination were proved by two experienced examiners. The average OCTA quality in all subjects measured by Heidelberg Spectralis II custom software was 38 ± 3 dB. Mean OCTA quality in patients with cornea verticilata was 38 ± 3 dB and 36 ± 4 dB in patients without cornea verticilata (*p* = 0.058). The obtained scans were analyzed using the Erlangen Angio Tool, which is as previously reported an application with high reliability and comparability of repeated macular scans [12].

**Fig. 2:**
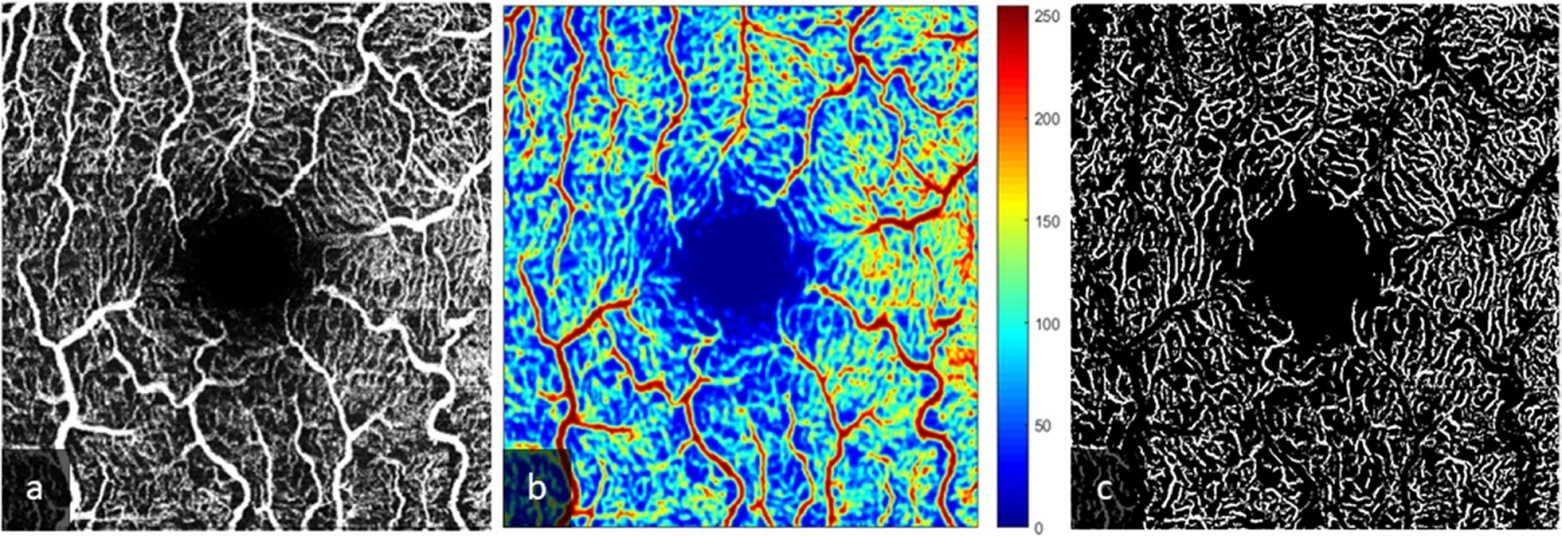
Images of superior vascular plexus (SVP) before and after segmentation. Original image of SVP (**a**), heatmap with large vessels marked in red (**b**) and image after segmentation and removal of large vessels (**c**)

It has a semi-automatic algorithm to segment and analyze the vessel density of the pre-defined region of interest (ROI) applying a series of algorithms to differentiate between tissue- and vessel-areas. The combination of the underlying Frangi vesselness filter and the Otsu Binarization with the possibility to mask out big vessels enabled the observation of vessel area density changes on a capillary level. The term 'Vessel Area Density (VAD)' was used in alignment with existing guidelines advocating for the standardization of optical coherence tomography angiography (OCTA) metrics [13]. Images of SVP before and after segmentation and removal of large vessels are presented in Fig. 2.

**Fig. 3:**
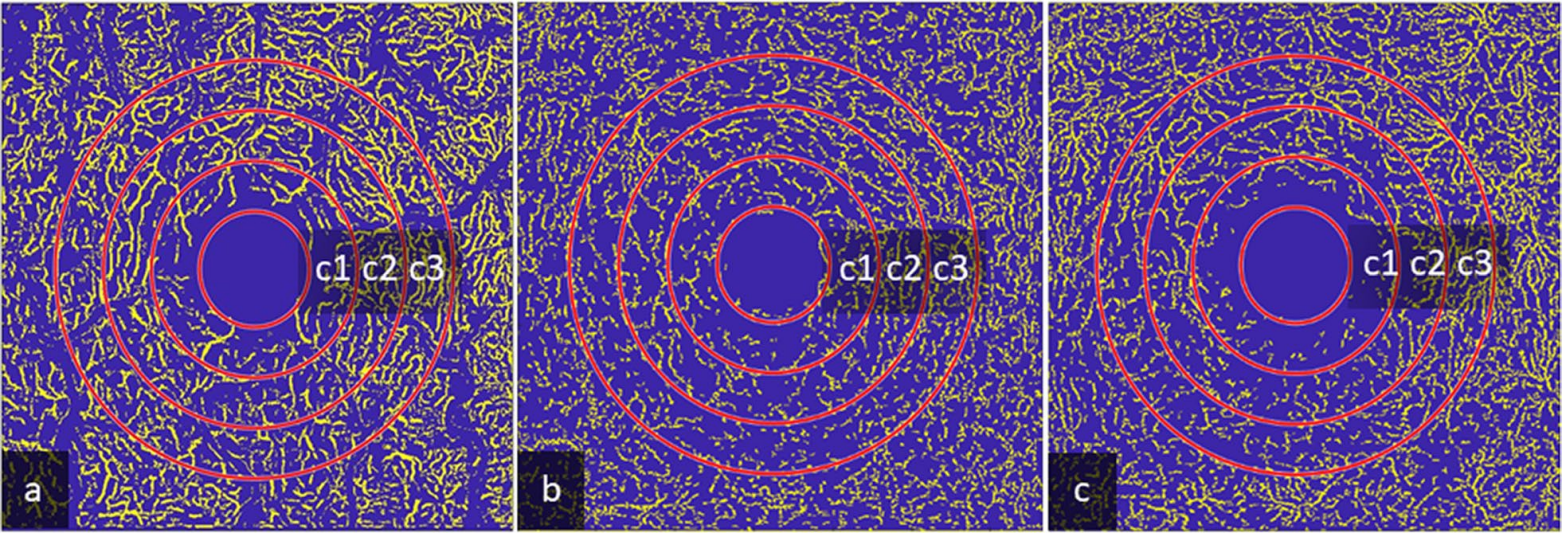
Segmentation of Macula OCTA scans. OCTA scans of the superficial vascular plexus with masking of bigger vessels measured from RNFL to inner boarder of the IPL [−] (**a**), intermediate capillary plexus measured from IPL [−] to outer boarder of IPL [+] (**b**), and deep capillary plexus measured from outer boarder of IPL [+] to OPL [+], including the OPL (**c**) with circular segmentation of macular sectors c1, c2, c3

OCTA scans of the superficial vascular plexus (SVP) were measured from retinal nerve fiber layer (RNFL) to inner border of inner plexiform layer (IPL) [−], intermediate capillary plexus measured from inner border of IPL [−] to outer border of IPL [+] and deep capillary plexus measured from outer border of IPL [+] to outer plexiform layer (OPL) including OPL [+]. The VAD was measured in full macular scans as well as 3 circular sectors (c1, c2 and c3) of SVP, ICP and DCP with the Erlangen Angio Tool and reported in percentages (%) (Fig. 3).

### Measurement of the Foveal Avascular Zone (FAZ)

**Fig. 4:**
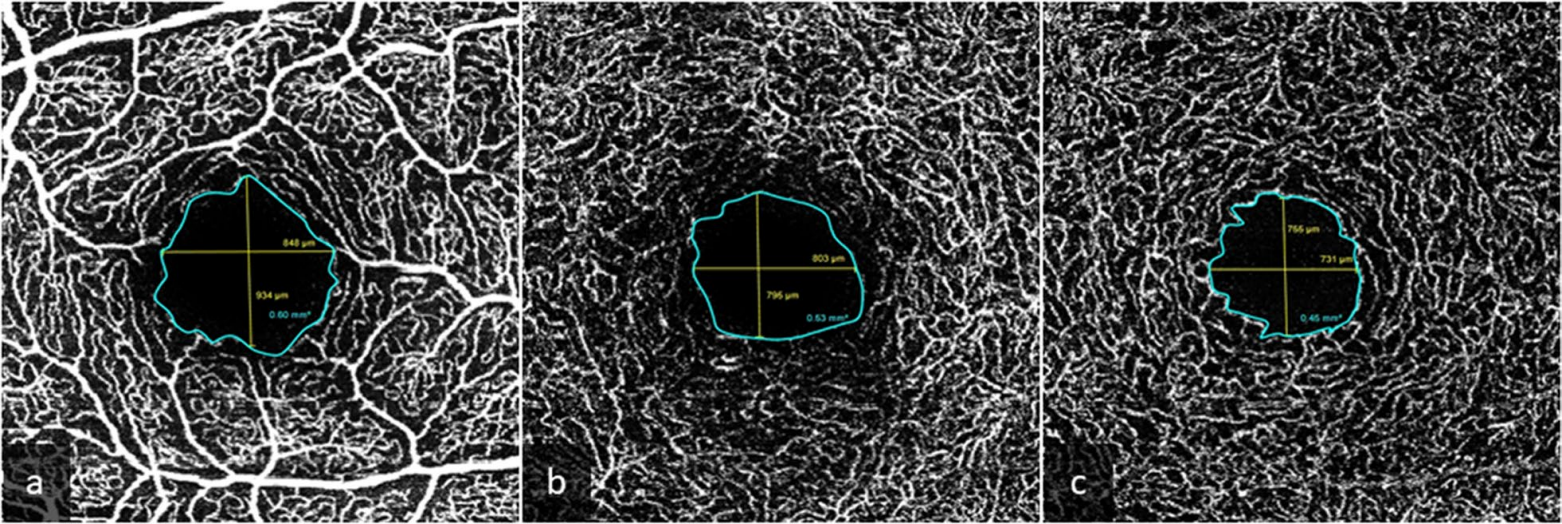
Foveal avascular zone area measurements done in SVP (**a**), ICP (**b**) and DCP (**c**) of the same patient

The Foveal Avascular Zone (FAZ) area (mm^2^) was delineated and measured manually in full-thickness scans, SVP, ICP and DCP using tools provided by Heidelberg Spectralis II custom software (Fig. 4).

### Statistical analysis

Statistical analysis was performed using SPSS v. 27.0 (IBM Corp., Armonk, NY, USA).

Kolmogorov–Smirnov and Shapiro–Wilk tests were used to analyze the normality of distribution of empirical data. Independent samples Student’s t-tests were used to compare and determine the equality of means in two normally distributed populations. The Mann Whitney U Test was used to compare the equality of two abnormally distributed populations. One-way ANOVA was performed to test the equality means of more than two normally distributed populations. Kruskal Wallis Test was used to evaluate variance in more than two abnormally distributed populations.

All means are presented with standard deviations (Mean ± SD). The Chi-square test of independence was used to analyze differences in two groups when the dependent variable was measured at a nominal level. Pearson and Spearman correlation coefficients were used to measure the linear correlation between variables. Results with a significance level of *p* < 0.05 were interpreted as statistically significant.

## Results

### Demographic und clinical data of Fabry disease patients

A total of 76 eyes of 38 Fabry disease patients (13 male and 25 female) and 48 eyes of 24 healthy controls (11 male and 13 female) were enrolled in the study. The mean age was 44.5 ± 15.2 years in FD patients and 35.3 ± 12.7 years in healthy controls. The demographic data and GLA mutations of patients are presented in Table 1.

**Table 1:** Alpha galactosidase gene (GLA) mutations and demographic data of Fabry disease patients

Mutation	Number of patients	Age, sex of patients
Intron 2; c*.*369 + 1G > T	2	21 M, 54 F
Exon 3; c.427G > A (ALA143Thr)	4	31 M, 47 M, 58 F, 59 F
Exon 3; (p.Ser126Gly)	1	49 F
Exon 4; c.610T > Cp. (W204R)	1	56 M
Exon 5; c.717-718 del AA or c.718-719 del AA fs 248X	2	48 F, 53 F
Exon 6; N320I *(*p*.*Asn320Ile*)*	1	31 M
Exon 6 Stop Codon; c.901C > T	1	44 F
Exon 7; c.1019G > A (p.W340*)	3	11 F, 13 M, 42 F
c.334C > T; p.R112C	2	25 M, 54 F
c.376A > Gp. (Ser126Gly)	1	34 M
c.427G > A	1	65 M
c.547 + 1G- > A	1	36 F
c*.*559A > G (p.Met187Val)	4	28 F, 35 F, 39 F, 60 M
c.717A > G p. (Ile239Met)	1	59 M
c.861G > A (p.Trp287*)	1	67 F
c.937G > T; p.D313Y	1	29 F
c*.*937G > T *(*p*.*Asp313Tyr*)*	3	24 F, 44 F, 64 M
c.1088G > A (p.Arg363His)	1	44 F
c.1087C > T (p.Arg363Cys)	2	40 F, 60 M
p.Asp313Tyr; D313Y	1	32 F
p.G361X	1	57 F
A215S	2	52 F, 72 F
D313Y	1	54 F

Cornea verticillata was observed in 55% (42/76 eyes) and vascular tortuosity in 26% (20/76 eyes). GLA activity was measured in 63% (24/38) patients. Within these, 79% (19/24) showed reduced and only 21% (5/24) showed normal activity. LysoGb3 plasma concentration was measured in 89% (34/38) patients, of which 59% (20/34) showed increased and 41% (14/34) showed reduced plasma concentration.

### Foveal avascular zone (FAZ)

Fovea plana was observed in one of the patients in the Fabry disease group. Therefore, this patient was excluded while performing foveal avascular zone measurements.

The average area of FAZ in full thickness scans in FD group was 0.23 ± 0.10 (0.03–0.51) and 0.25 ± 0.07 (0.12–0.37) mm^2^ in healthy controls. No statistically significant difference in FAZ was observed between the groups (*p* = 0.2). The average area of FAZ in SVP, ICP and DCP did not significantly differ between FD patients and healthy controls (*p* > 0.05). Correlation analysis showed no significant correlation between subjects age and FAZ area in full-thickness, SVP, ICP and DCP scans (*p* > 0.05; Pearson correlation coefficient from − 0.11 to 0.09). The extended results comparing FAZ between FD patients and healthy controls are presented in Table 2.

**Table 2:** Foveal avascular zone and mean vessel area density in Fabry disease group vs. controls

	FD (n = 74–76); mean ± SD	Controls (n = 48); mean ± SD	95% CI [lower; upper]	*p* value
*FAZ area (mm*^*2*^*)*				
Full thickness	0.23 ± 0.10	0.25 ± 0.07	[− 0.05; 0.02]	0.205**
SVP	0.36 ± 0.12	0.35 ± 0.08	[− 0.02; 0.05]	0.795**
ICP	0.22 ± 0.11	0.24 ± 0.069	[− 0.05; 0.01]	0.145*
DCP	0.40 ± 0.16	0.36 ± 0.07	[− 0.01; 0.08]	0.121**
*VAD (%)*				
SVP	49.37 ± 11.02	26.51 ± 6.18	[19.79; 25.92]	< 0.001**
ICP	29.57 ± 7.44	20.17 ± 4.38	[7.30; 11.75]	< 0.001**
DCP	32.25 ± 8.81	21.71 ± 5.08	[8.07; 13.01]	< 0.001**

*Student t-test, **Mann Whitney U Test

Bold indicates statistical significance (*p* < 0.05)

### Vessel area density (VAD)

The mean VAD in macula scans of SVP, ICP and DCP in FD group was higher than in the control group (49.4 ± 11.0 vs. 26.5 ± 6.2, 29.6 ± 7.4 vs. 20.2 ± 4.4, 32.3 ± 8.8 vs. 21.7 ± 5.1 respectively, *p* < 0.001). Additionally, the average VAD values were significantly higher in FD group when compared to healthy controls in all circular sectors in all layers (*p* < 0.05; data not shown). The extended results comparing VAD between FD patients and healthy controls are presented in Table 2.

Correlation analysis between subject age and VAD revealed no significant correlation between age and VAD in SVP and DCP (*p* > 0.05; Spearman’s rho from − 0.17 to 0.04). A weak negative correlation was observed between subjects age and VAD in ICP (*p* < 0.05; Spearman’s rho − 0.20).

### Clinical ophthalmological findings and FAZ and VAD

FAV and VAD was compared between FD patients with cornea verticillata (CV), FD patients without CV and healthy controls as well as FD patients with vascular tortuosity, FD patients without vascular tortuosity and healthy controls. There was no statistically significant difference in FAZ between the three groups (*p* > 0.05). When comparing eyes with and without CV a significantly higher FAZ area in SVP was observed in patients without CV and healthy controls (*p* < 0.05). A significant difference in VAD was observed in all examined layers between patients with and without CV and controls (*p* < 0.001).

No significant difference in VAD in full SVP scans as well as all SVP sectors was observed between patients with and without CV. However, when comparing patients with CV to controls and patients without CV in SVP the variation between these groups was significant (*p* < 0.05). FD eyes with or without CV had a higher VAD when compared to controls.

Compared to controls both FD eyes with CV (*p* < 0.01) and FD eyes without CV (*p* < 0.05) had a higher VAD in all layers than healthy control eyes. Extended results are presented in Table 3.

**Table 3:** FAZ and VAD in patients with and without cornea verticillata compared to controls

	FD eyes with cornea verticillata (n = 40–42); mean ± SD	FD eyes without cornea verticillata (n = 34); mean ± SD	Controls (n = 48); mean ± SD	*p* value
*FAZ area (mm*^*2*^*)*				
Full thickness	0.22 ± 0.09	0.26 ± 0.11	0.25 ± 0.07	0.196
SVP	0.35 ± 0.11	0.38 ± 0.13^b^	0.35 ± 0.08^b^	0.780
ICP	0.19 ± 0.10	0.24 ± 0.11	0.24 ± 0.069	0.142
DCP	0.38 ± 0.10^a^	0.41 ± 0.21	0.36 ± 0.07^a^	0.677
*VAD (%)*				
SVP	50.95 ± 8.74^a^	47.41 ± 13.19^b^	26.51 ± 6.18^ab^	**< 0.001**
ICP	31.70 ± 6.63^ac^	28.94 ± 7.62^bc^	20.17 ± 4.38^ab^	**< 0.001**
DCP	35.30 ± 6.99^ac^	28.47 ± 9.44^bc^	21.71 ± 5.08^ab^	**< 0.001**

Kruskal Wallis Test, Bold indicates statistical significance (*p* < 0.05)

^a ^*p* < 0.01 using Mann–Whitney U Test between eyes with cornea verticillata and controls

^b ^*p* < 0.05 using Mann–Whitney U Test between eyes without cornea verticillata and controls

^c ^*p* < 0.01 using Mann–Whitney U Test between eyes with and without cornea verticillata

No difference in FAZ was observed between patients with or without vascular tortuosity and healthy controls (*p* > 0.05). When comparing VAD between the three groups a significant difference in VAD was observed in all layers (*p* < 0.001).

There was no difference in VAD in patients with and without vascular tortuosity. However, a significantly higher VAD was observed in patients with vascular tortuosity compared to controls (*p* < 0.03) and in patients without vascular tortuosity compared to controls (*p* < 0.001) in full SVP, ICP and DCP scans. Extended results of FAZ and VAD measurements in patients with and without vascular tortuosity as well as controls are presented in Table 4.

**Table 4:** FAZ and VAD in patients with and without vascular tortuosity compared to controls

	FD eyes with vascular tortuosity (n = 20); mean ± SD	FD eyes without vascular tortuosity (n = 54–56); mean ± SD	Controls (n = 48); mean ± SD	*p* value
*FAZ area (mm*^*2*^*)*				
Full thickness	0.24 ± 0.08	0.24 ± 0.11	0.25 ± 0.07	0.378
SVP	0.37 ± 0.10	0.36 ± 0.13	0.35 ± 0.08	0.812
ICP	0.22 ± 0.09	0.21 ± 0.11	0.24 ± 0.069	0.214
DCP	0.37 ± 0.11	0.41 ± 0.21	0.36 ± 0.07	0.829
*VAD (%)*				
SVP	50.15 ± 9.05^a^	49.08 ± 11.71^b^	26.51 ± 6.18^ab^	**< 0.001**
ICP	32.18 ± 5.62^a^	28.64 ± 7.82^b^	20.17 ± 4.38^ab^	**< 0.001**
DCP	33.75 ± 6.78^a^	31.71 ± 9.42^b^	21.71 ± 5.08^ab^	**< 0.001**

Kruskal Wallis Test, Bold indicates statistical significance (*p* < 0.05)

^a ^*p* < 0.03 using Mann–Whitney U Test between eyes with vascular tortuosity and controls

^b ^*p* < 0.001 using Mann–Whitney U Test between eyes without vascular tortuosity and controls

### Foveal avascular zone, vessel area density and enzyme activity/concentration

Out of cases where enzyme activity was tested, 90% (34/38) of subjects with cornea verticillata had an increased LysoGb3 plasma concentration. From subjects without CV LysoGb3 was only increased in 20% (6/30) of cases. LysoGb3 concentration was increased in cases with CV when compared with subjects without CV (*p* < 0.001). There was no significant difference in the distribution of subjects with decreased GLA activity in subjects with and without cornea verticillata (71% (20/28) vs. 90% (18/20) respectively, *p* = 0.113).

80% (16/20) of subjects with vascular tortuosity had an increased LysoGb3 concentration meanwhile only 50% (24/48) of subjects without vascular tortuosity had increased LysoGb3 concentration (*p* = 0.02).

A larger FAZ area in full-thickness scans and ICP was observed in patients with decreased GLA activity (*p* < 0.04). There was no significant difference in FAZ area according to LysoGb3 plasma concentration (*p* > 0.05). Patients with increased plasma concentration of lysoGb3 had a higher VAD in DCP when compared to patients with normal lysoGb3 concentration (*p* = 0.04). Extended results of FAZ and VAD between patients with normal or pathological enzyme activity are presented in Table 5.

**Table 5:** FAZ and VAD in Fabry disease patients with normal or altered enzyme activity

	Normal GLA activity (n = 10); mean ± SD	Decreased GLA activity (n = 38); mean ± SD	*p* value	Normal lysoGb3 concentration (n = 28); mean ± SD	Increased lysoGb3 concentration (n = 40); mean ± SD	*p* value
*FAZ area (mm*^*2*^*)*						
Full thickness	0.18 ± 0.07	0.24 ± 0.09	**0.037**	0.25 ± 0.13	0.23 ± 0.08	0.950
SVP	0.33 ± 0.10	0.35 ± 0.12	0.736	0.37 ± 0.15	0.36 ± 0.11	0.822
ICP	0.15 ± 0.08	0.22 ± 0.09	**0.026**	0.22 ± 0.14	0.22 ± 0.08	0.324
DCP	0.33 ± 0.10	0.36 ± 0.14	0.644	0.41 ± 0.22	0.38 ± 0.10	0.373
*VAD (%)*						
SVP	52.71 ± 7.70	51.08 ± 10.33	0.556	46.43 ± 13.76	50.41 ± 8.80	0.247
ICP	31.36 ± 4.84	29.57 ± 7.82	0.644	27.00 ± 8.38	30.69 ± 6.47	0.065
DCP	37.10 ± 6.51	32.45 ± 8.28	0.120	28.63 ± 9.76	34.35 ± 7.13	**0.015**

Mann Whitney U Test, Bold indicates statistical significance (*p* < 0.05)

## Discussion

Our study showed no significant difference in FAZ area of Fabry patients when compared to normal controls. Other OCTA studies that evaluated FAZ in Fabry disease found either that the FAZ was larger in FD when compared with healthy controls or that these measurements were the same in both groups. Two studies found that FAZ was larger in both the superficial and deep capillary plexus in FD [14, 15]. Cakmak et al. found that FAZ in FD was larger than in healthy controls [16]. Meanwhile two other studies found no significant difference in FAZ between FD patients and healthy subjects [17, 18]. The results of these studies in combination with our data support the assumption that FAZ alone is not a reliable biomarker for vascular changes due to the very high variability of FAZ in Fabry patients and in healthy controls [19, 20]

A higher VAD was observed in all vascular layers of the macula in FD patients when compared to healthy controls. This increased VAD in FD could be a result of retinal vasodilation, excess release of reactive oxygen species (ROS) and accumulation of glycosphingolipids [8].

In contrast to our findings several other studies reported a decreased vascular density in Fabry disease [17, 18, 21]. Lin et al. reported a decreased vessel length density and vessel perfusion density in the superior vascular plexus [18]. Bacherini and colleagues described a reduction of vessel density in whole and outer SVP as well as whole, inner and outer DCP [17]. Cakmak et al. reported a reduction of vessel density in the fovea of both superior and deep capillary plexuses which is supported by their findings of increased FAZ in FD. However, no significant difference in vascular density in other segments of superior and deep capillary plexuses was found [16]. In a long-term follow-up of 26 eyes, we observed a decrease of VAD in patients with Fabry disease over time, which points to vascular remodelling during the course of the disease [22].

In contrast to other studies Atiskova et al. reported no difference in perifoveal vessel density when comparing FD patients with controls [23]. A study by Dogan et al. reported a reduction of deep capillary vascular density in FD but no difference in superficial capillary vascular density in FD and controls [21]. However, other studies in turn reported an increase in VAD [14, 24]. Minnella et al. reported an increase in vessel density in the superior vascular plexus and no difference in vessel density in DCP between FD patients and healthy subjects [14]. Cennamo et al. found an increase of vascular density in DCP but decrease of vascular density in SVP which could be due to examining patients in early stages of vascular remodelling [24]. Inconsistent findings among studies may also a result of inconsistencies in methodology between different studies. We believe that discrepancies between results could occur depending on the stage of the disease.

In addition to analyzing FAZ and VAD, we explored the association of OCTA data with clinical ocular findings in FD patients. We observed that the increase of VAD in intermediate and deep vascular plexuses was associated with clinical presence of cornea verticillata. This supports our hypothesis that patients with cornea verticillata have an increased accumulation of glycophospholipids not only in the cornea but also in the vessels of the deeper retinal layers which leads to vascular hyperperfusion and increased VAD. These findings are supported by another study which observed that patients with cornea verticillata had a higher whole image deep capillary vessel area density when compared to patients without cornea verticillata [21].

We strongly believe that cornea verticillata could be used as a predictive biomarker for changes in VAD and disease progression. We suggest that combination of cornea verticillata and increased VAD may be considered as a diagnostic biomarker for Fabry disease if more studies with large sample sizes report on similar results. However, at the moment due to the discrepancies in studies on VAD this claim cannot be supported without further investigations.

Surprisingly, there was no significant difference in VAD between patients with and without vascular tortuosity but both groups have a significantly higher VAD when compared to healthy controls. This suggest that the vascular remodelling occurs in all retinal layers in both FD patients with and without vascular tortuosity and changes in VAD can occur before visible clinical changes to the retinal vasculature. We believe that the higher VAD in both groups is related either to the tortuosity or to hyperpermeability of the blood vessels. This underlines the importance of an early baseline OCTA examination after the diagnosis of FD, which could be helpful in tracking the disease progression over time.

In a subgroup analysis of patient with and without vascular tortuosity we found increased lysoGb3 concentrations in both groups which reached up to 80% in the FD group with vascular tortuosity. When looking into the laboratory findings in FD we found that patients with an increased plasma concentration of lysoGb3 had a significant increase in VAD in DCP compared to patients with a normal concentration of lysoGb3. Opposite to our findings Wiest et al. previously reported an inverse association of VAD in superficial and deep capillary plexuses with lysoGb3 plasma concentration [25].

The association of presence of vascular tortuosity and increased plasma concentrations could be explained by the increased concentration of lyso-Gb3 which promotes globotriaosylceramide storage and induces proliferation of smooth muscle cells [26]. Abnormally proliferating vascular smooth muscle cells express adhesion molecules and produce cytokines that lead to the influx of inflammatory cells and initiates the cascade of inflammation in the vessel wall [27, 28]. These cytokines also induce vascular cell growth, promote adhesion of immune cells to endothelial cells and cause an increase in vascular permeability [29]. Arterial remodelling results in thickened, less compliant vascular walls, this in association with hyperdynamic circulation, can increase angiotensin-II levels by triggering an upregulation of local renin angiotensin systems [28, 30]. Angiotensin-II initiates also an inflammatory cascade that increases production of ROS and nuclear factor-kappa B, which mediates transcription and gene expression and increases adhesion molecules and chemokines [31]. The inflammatory process and oxidative stress progressively weakens the vessel wall as it activates protease-mediated extracellular matrix degradation and apoptosis of smooth muscle cells, which in culminates in dilatation and aneurysm formation in the course of the disease [27, 28].

Knowing this cascade of vascular remodelling in Fabry disease it is comprehensible that weakening of the blood vessel walls cause dilatation of these vessels in the course of the disease. We hypothesize that the initial vascular damage starts in the endothelium of the capillaries of deeper retinal vascular layers due to a single layer vessel wall structure. Because of a lack of smooth muscle cells in the walls of the capillaries in the deeper vascular layers the endothelial changes could be detected in the capillaries in the earlier stage of the disease.

While large vessels are encircled by smooth muscle cells, capillaries are instead surrounded by muscle-like cells called pericytes [32]. SVP consist of a mixture of large and small vessels whereas ICP and DCP consists of thin layers of capillaries arranged in lobular patterns [33]. Because of these structural differences the deep vascular layers are susceptible to vascular damage earlier that the super vascular plexus which has larger blood vessels with more smooth muscle cells. This explains the discrepancies between various studies because the patients could be examined in different stages of the disease. We hypothesize that significant changes in the arterioles in the superior vascular plexus occur in later stages of the disease and therefore measuring VAD in OCTA scans has great potential to be used as a retinal biomarker in grading vascular changes in Fabry disease. This is backed up by an OCTA study that observed an increase in vascular density in whole image, foveal and parafoveal deep capillary plexus and a decrease in vascular density in whole image, foveal and parafoveal superior capillary plexus [24]. Because patients with cornea verticillata were excluded from the study these findings most likely pertain only to patients in early stages of the disease. This supports our theory that cornea verticillata is a good predictive biomarker for disease progression. Due to the fact that more than half of patients in our study had clinical signs of cornea verticillata and increased VAD in all retinal layers we believe that this represent a high disease activity in our cohort.

The strength of our study is the exploration of various clinical and laboratory parameters as possible biomarkers of vascular remodelling in Fabry disease. This further contributes to a better understanding of this disorder and highlights an importance of an intradisciplinary approach in monitoring of FD. Another strength of our study is the big sample size of Fabry disease patients whose retinal microvasculature was examined using OCTA using high quality imaging as well as manually determining the FAZ. According to our literature analysis it is one of the largest OCTA studies pertaining Fabry disease. To the authors knowledge it is the largest study of this kind that evaluates the correlation between clinical ocular findings with FAZ and VAD. One of the strengths of our study is the comparison of OCTA findings between Fabry disease patients and healthy controls.

However, our study has potential limitations. The axial length was not measured and the FAZ area is not adjusted to axial length. To adjust for this, we have not included patients with a refractive error of more than 3.5 dpt. We also did not examine the repeatability of our measurements. However, the repeatability and reproducibility of VAD measurements done in SVP, ICP, DCP scans performed with Spectralis OCT II and analyzed with the semiautomated vessel density software EA-Tool was previously proved [12].

## Conclusions

Our findings suggest that the vascular remodeling in FD affects all retinal vascular layers. Due to the vascular structural differences of the retinal vessels in inner and outer retinal layers the deeper layers seem to be more susceptible to vascular damage earlier in the course of the disease when compared to superior vascular layers. We suggest that increased plasma concentration of lysoGb3 and increase in VAD in deep vascular layers could be reliable biomarkers of activity of the disease. Cornea verticillata could be used as a predictive biomarker for VAD changes and disease progression. The combination of cornea verticillata and increased VAD may serve as a diagnostic biomarker for Fabry disease, however due to the discrepancies in VAD values in various studies, further research has to be done to address this claim. In the future more longitudinal OCTA studies is needed to establish a grading system for vascular changes based on VAD in different vascular retinal layers during various stages or different phenotype of Fabry disease.

## Data Availability

The datasets used and/or analysed during the current study are available from the corresponding author on reasonable request.

## Abbreviations

CV: Cornea verticillata
DCP: Deep capillary plexus
FAZ: Foveal avascular zone
FD: Fabry disease
Gb_3_: Globotriaosylceramide
GLA: Alpha galactosidase
ICP: Intermediate capillary plexus
IPL: Inner plexiform layer
Lyso-Gb-3: Globotriaosylsphingosine
VAD: Vessel area density
OCTA: Optical coherence tomography angiography
OPL: Outer plexiform layer
RNFL: Retinal nerve fiber layer
ROS: Reactive oxygen species
SVP: Superficial Vascular Plexus
