# Correction: Correlation of retinal vascular characteristics with laboratory and ocular findings in Fabry disease: exploring ocular diagnostic biomarkers

**DOI:** 10.1186/s13023-025-04162-9

**Published:** 2026-05-08

**Authors:** Migle Lindziute, Jessica Kaufeld, Karsten Hufendiek, Ingo Volkmann, Dorothee Brockmann, Sami Hosari, Bettina Hohberger, Christian Mardin, Carsten Framme, Jan Tode, Katerina Hufendiek

**Affiliations:** 1 University Eye Hospital, Hannover Medical School, Hannover, Germany; 2 Division of Nephrology, Center for Internal Medicine, Hannover Medical School, Hannover, Germany; 3 Department of Surgery, Friedrich-Alexander-University of Erlangen-Nuremberg, Erlangen, Germany; 4 Department of Ophthalmology, Friedrich-Alexander-University of Erlangen-Nuremberg, Erlangen, Germany

**Correction to: Orphanet J Rare Dis 18, 314 (2023)**

10.1186/s13023-023-02932-x.

Following publication of the original article [1], the authors reported that the names and family names of the authors Christian Mardin and Jan Tode had been mistakenly reversed. The corrected names provided in this Correction is accurate.

Additionally, the authors identified imprecise use of terminology regarding Lyso-Gb3 concentration and GLA enzyme activity. In several instances, these were incorrectly referred to as “LysoGb3 activity” and “GLA concentration”. This has also been corrected.

The original article [1] has been updated.
